# Social equity in Ethiopian infants’ breastfeeding and complementary feeding practices: a birth cohort study

**DOI:** 10.1186/s12887-025-06105-6

**Published:** 2025-10-09

**Authors:** Amare Tariku, Kassahun Alemu, Joanna Schellenberg, Tanya Marchant, Della Berhanu, Seblewengel Lemma, Atkure Defar, Theodros Getachew, Zewditu Abdissa, Tadesse Guadu, Solomon Shiferaw, Girum Taye, Meseret Zelalem, Lars Åke Persson

**Affiliations:** 1 Institute of Public Health, College of Medicine and Health Sciences, University of Gondar, Gondar, Ethiopia; 2 Department of Disease Control, London School of Hygiene & Tropical Medicine, London, UK; 3 Health System and Reproductive Health Research Directorate, Ethiopian Public Health Institute, Addis Ababa, Ethiopia; 4 School of Public Health, Addis Ababa University, Addis Ababa, Ethiopia; 5 Maternal, Child & Adolescent Health Service Lead Executive Office, Federal Ministry of Health, Addis Ababa, Ethiopia

**Keywords:** Breastfeeding, Complementary feeding, Social equity, Performance monitoring for action ethiopia

## Abstract

**Introduction:**

Monitoring social equity in infant feeding is essential to track countries’ progress towards global child nutrition and survival targets. We aimed to examine social equity in Ethiopian infants’ early initiation of breastfeeding, exclusive breastfeeding at five months, and quality of complementary foods at 12 months.

**Methods:**

This study was a secondary analysis of the Ethiopia Performance Monitoring for Action panel data, prospectively collected from July 2020 to August 2021. A total of 1,850 infants were followed from birth to 12 months in five Ethiopian regions: Addis Ababa City Administration, Oromia, Amhara, Afar, and Southern Nations, Nationalities, and Peoples Regions. We analyzed social equity in infants’ early initiation of breastfeeding, exclusive breastfeeding at five months of age, and dietary diversity at 12 months of age, calculated slope and concentration indices and using mixed-effect regression models.

**Results:**

Most infants started breastfeeding within one hour of birth (67%, 95% CI 63, 71) and were exclusively breastfed (69%, 95% CI 67, 71) at five months of age. Few (16%, 95% CI 13, 19) infants aged 12 months had complementary foods meeting the minimum quality criterion, i.e., from five or more food groups. Half (49%, 95% CI 44, 55) of infants aged 12 months consumed sugary foods or beverages. There was no inequity in early initiation and exclusive breastfeeding practices by mothers’ education and household wealth levels. There was social inequity in infants’ dietary diversity at 12 months of age, favoring educated (slope index: 0.368, p-value < 0.001) and better-off (concentration index: 0.350%, p-value < 0.001) families.

**Conclusions:**

The high coverage of early initiation of breastfeeding and exclusive breastfeeding at five months of age were equitably distributed by mothers’ education and household wealth. Few 12-month-old infants had a diverse diet, while half consumed sugary foods or beverages. The quality of complementary food was inequitable, favoring better-off and more educated families. Ensuring optimal access to infant feeding support for socially disadvantaged families is critical to improving the quality of complementary food and maintaining or further increasing appropriate breastfeeding.

## Introduction

There are well-described social disparities in child undernutrition and mortality in low- and middle-income countries [1, 2]. The World Health Organization and UNICEF endorsed infant and young child feeding as a critical strategy for promoting child growth and survival and attaining the Sustainable Development Goals (SDG) of ending child undernutrition and reducing mortality [3, 4]. Breast milk is an unparalleled food, offering balanced nutrients crucial for infants’ optimal physical and cognitive development [5]. The immune properties of human breast milk are critical for the protection against infectious diseases, such as diarrhea and pneumonia [6]. The initiation of breastfeeding within one hour of birth further enhances newborns’ immunity and health, promotes mother-infant bonding, and improves the likelihood of continued exclusive breastfeeding [7, 8]. At around six months of age, infants should gradually be introduced to complementary foods, so that the one-year-old infant receives for a diverse diet with continued breastfeeding and components from a minimum of five food groups, that add further energy, protein and micronutrients [9].

Social inequities in breastfeeding and complementary feeding, favoring socially advantaged children, hinder countries’ progress toward attaining child nutrition- and survival-related SDG targets [10]. Ensuring equitable access to quality infant feeding services is central to reaching SDGs through supporting socially disadvantaged women and their children [11].

Only half of infants from low- and middle-income countries were put to the breast within one hour of birth, 32% were exclusively breastfed at five months, and less than one-fifth (17%) of infants aged 12 months had complementary foods meeting the minimum diet diversity, i.e., from five or more food groups [12, 13]. The low coverage of appropriate infant feeding practices showed wealth- and education-based social disparities in low- and middle-income countries. The coverage of early breastfeeding initiation was higher among infants of educated and better-off mothers in sub-Saharan African countries [14]. Exclusive breastfeeding 0–6 months was more common among infants with more educated and affluent households in these countries [15]. Dietary diversity and animal source food consumption (dairy products, flesh foods, and eggs) have been reported to be inequitably distributed, with diet diversity being more frequent among infants of better-off families [12].

The Ethiopia Health Sector Transformation Plan-II stated equitable access to quality child feeding and nutrition services as one of the transformation agendas to reduce undernutrition and mortality in children under five years [16]. We have previously reported that in 2020–2021, most Ethiopian infants initiated breastfeeding within one hour of birth (67%) and were exclusively breastfed at five months of age (69%) [17]. However, less than one-fifth of infants aged 12 months had complementary foods meeting the minimum dietary diversity. Regular monitoring of social disparities in infant feeding practices helps to inform efforts to promote breastfeeding and complementary feeding and target vulnerable groups in need of special feeding support. There are few Ethiopian studies of social equity in early breastfeeding initiation, exclusive breastfeeding, and complementary feeding practices among infants and young children [16, 18]. None of these studies assessed the absolute or relative social equities in early initiation and exclusive breastfeeding coverage and the quality of complementary food. Thus, we aimed to assess social equity in infants’ early initiation of breastfeeding, exclusive breastfeeding at five months, and dietary diversity at 12 months.

## Methods

### Study setting, design, and population

Ethiopia is a low-income country with an average per capita income of $1,020 [19]. It is Africa’s second most populous country, with an adult literacy rate of 51%. 84% of the Ethiopian population is living in rural areas. This birth cohort study was a component of the Ethiopia Performance Monitoring for Action (PMA) panel study carried out by Addis Ababa and Johns Hopkins Universities from July 2020 to August 2021 to evaluate the coverage and comprehensiveness of the continuum of Reproductive, Maternal and Newborn Health services from pregnancy to one year after birth [20]. Our analysis was based on samples from five regions representing 85% of the Ethiopian population: Oromia, Amhara, and Southern Nation, Nationalities, and Peoples regions (predominantly agrarian regions), the pastoralist Afar region, and the urban Addis Ababa.

### Study population and sampling

All pregnant women (15–49 years) or those within six weeks postpartum who were regular members of households or living with parents for delivery or postpartum care were eligible for the study. Eligible women were selected using multistage cluster sampling. A sampling frame comprising a list of clusters or enumeration areas (EAs) was obtained from the Ethiopian Central Statistical Agency. The EAs were selected with probability proportional to the size of the region. Women who fulfilled the eligibility criteria were invited to participate in the study. Because of armed conflict, the data collection stopped in the Tigray region in November 2021. Therefore, our secondary analysis of the PMA data was based on the five Ethiopian regions, excluding Tigray.

### Data collection

Field workers took informed consent from eligible women and collected PMA data with a standardized questionnaire. Two weeks of training were given to regional coordinators, field supervisors, and data collectors on study aim, protocol, and interview techniques. Data collectors used interview guides and received regular supervision. Data were electronically collected using Open Data Kit software. Data collectors made regular phone calls to check if a woman had given birth. After that, the first, second, and third follow-up interviews were conducted at about six weeks, six months, and twelve months after birth.

In this study, we used the World Health Organization infant feeding measurement approach [4]. Based on an interview around six weeks postpartum, the time of breastfeeding initiation was assessed. We defined early breastfeeding initiation as infants put to their mothers´ breasts within one hour of birth. Interviewers asked mothers about whether they gave breast milk in the 24 h before the survey at the first, second, and third follow-up interviews. They asked whether any other food had been introduced and, if so, at what age in months this happened. The coverage of exclusive breastfeeding was calculated as the proportion of infants fed only breast milk at five months of age. Continued breastfeeding at six weeks, six months, and twelve months was ascertained when infants were fed breast milk in the 24 h before the respective follow-up interviews. A 24-hour recall was used to assess infants’ complementary feeding. The minimum diet diversity was defined as the percentage of infants aged 12 months with complementary feeding, including five or more food groups, in the 24 h before the interview. Vitamin A-rich food consumption was illustrated as the proportion of infants aged 12 months who ate vitamin A-rich foods, i.e., organ meat, egg, dairy, vitamin A-rich vegetables or tubers, green leafy vegetables, or yellow-orange fruits. Iron-rich food intake was shown as the percentage of infants aged 12 months who consumed organ meat, meat, fish, or seafood in the day before the survey. Animal protein-rich food intake comprised infants’ flesh, egg, or dairy consumption. The consumption of energy-dense and low‐ micronutrient‐containing foods was assessed. Feeding practices were shown as the proportion of infants who drank sweet beverages or ate sugary or savoury foods in the 24 h before the survey. Sweet beverages included all homemade or commercially produced and packed drinks flavoured with sweeteners or to which sugar was added. Sugary or savoury foods comprised homemade or commercially produced and packed sugary and fried foods, including fried samosa, donuts, chips, cake, pastries, sweet biscuits, or candies. Detailed information about the study population, design, and data collection is available elsewhere [20].

### Analysis

Mothers’ characteristics and infant feeding practices were summarized using descriptive statistics, i.e., proportion, mean, and median. Sampling weights were generated as the inverse probability of enumeration areas and household selections. We used sampling weights in the analyses to ensure that the results represented women in the study regions. Analyses of infant feeding practices, socio-economic and other characteristics were adjusted for clustering.

We employed absolute and relative equity analyses to display any presence of socio-economic and educational disparities in infants’ early initiation of breastfeeding, exclusive breastfeeding at five months, and dietary diversity at one year. These social equity analyses were adjusted for clustering. The absolute inequity in infant feeding indicators was shown as the crude difference in appropriate infant feeding practices between the highest and lowest household wealth quintiles and the mothers’ education status. Relative differences in appropriate breastfeeding and complementary feeding indicators by socio-economic or educational status were illustrated using equiplots. The slope index of inequity (SII) was used to examine absolute inequity considering household health quintile and mothers’ education status. The relative index of inequity was shown using ratios and the concentration index (CIX). The ratio was calculated by dividing infant feeding practices between the highest and lowest wealth quintile or education levels. The coefficients and the 95% Confidence Intervals (CI) of the CIX and SII were used to show the strength of association and statistical significance of socio-economic and educational inequities in infant feeding practices. Outcomes were considered statistically significant at a p-value less than 0.05.

A mixed-effect binary logistic regression was used to examine the associations between socio-demographic factors, other covariates, and infants’ early initiation of breastfeeding, exclusive breastfeeding at five months, and receiving foods with a minimum level of dietary diversity. A random-effect model intended to account for intra-cluster correlation in infant feeding outcomes. We analyzed Crude Odds Ratios (COR) and Adjusted Odds Ratios (AOR) with a corresponding 95% CI.

## Results

### Participation

At baseline assessment, 27,722 women of reproductive age were screened for eligibility. A total of 2,585 women were eligible, i.e., either pregnant or within six weeks after birth. The first follow-up assessment was completed for 2304 newborns, and 1,850 infants aged 12 months had complete data from three follow-up interviews. Data were missing for 454 infants because of unavailability at follow-up (*n* = 303), incomplete data (*n* = 74), or infant death (*n* = 72).

### Socio-demographic characteristics of mothers

We included 1,850 infants in the analyses with three follow-up interviews. Most of their mothers were from rural areas and less than one-fifth had attended secondary school or above (Table 1).

**Table 1:** Socio-demographic characteristics of mothers and infants who completed a one-year postpartum follow-up, PMA Ethiopia panel study, July 2020 to August 2021 (*n* = 1,850)

Characteristics	Unweighted *n* (%)	^A^Weighted % (95% CI)
Region		
Afar	178 (10)	2 (1, 3)
Amhara	413 (22)	24 (21, 27)
Oromia	539 (29)	46 (41, 51)
Southern Nations, Nationalities, and Peoples	506 (27)	24 (20, 28)
Addis Ababa	214 (12)	4 (3, 5)
Residence		
Urban	640 (35)	21 (16, 27)
Rural	1,210 (65)	79 (73, 84)
Household size (*n* = 1,849)		
One to four	961 (52)	49 (45, 53)
Five to six	561 (30)	31 (29, 34)
Seven to sixteen^B^	327 (18)	20 (17, 24)
Household wealth quintiles (*n* = 1,849)		
1 (Poorest)	353 (19)	21 (16, 27)
2	297 (16)	20 (17, 23)
3	312 (17)	21(18, 24)
4	331 (18)	19 (16, 24)
5 (Wealthiest)	556 (30)	19 (14, 24)
Mothers age (in years) (*n* = 1,849)		
15–19	145 (8)	9 (7, 10)
20–24	433 (23)	23 (21, 26)
25–29	624 (34)	33 (30, 35)
30–34	364 (20)	20 (17, 22)
35–47	283 (15)	17 (15, 19)
Marital status (*n* = 1,849)		
Currently married^C^	1,817 (98)	98 (97, 99)
Currently unmarried^D^	32 (2)	2 (1, 3)
Education (*n* = 1,849)		
No schooling	753 (41)	43 (38, 48)
Primary school	671 (36)	40 (36, 44)
Secondary school or above	425 (23)	17 (14, 21)
Parity^E^ (*n* = 1,846)		
1	321 (17)	16 (14, 18)
2	427 (23)	21 (19, 24)
3–4	550 (30)	29 (27, 31)
≥ 5	548 (30)	34 (30, 38)
Number of antenatal care visits (*n* = 1,848)		
0	487 (26)	23 (19, 29)
1	122 (7)	9 (6, 12)
2	199 (11)	13 (11, 16)
3	348 (19)	22 (19, 25)
≥ 4	691 (37)	33 (28, 38)
Place of delivery (*n* = 1,849)		
Health facility	1,097 (59)	55 (49, 61)
Home	752 (41)	45 (39, 51)

^A^ Analysis adjusted for enumeration area and household sampling weights

^B^18 households had 11-16 members

^C^43 Living with a partner

^D^Divorced, never married and widowed; and

^E^14 mothers had 10-14 parity history

^A^Analysis adjusted for enumeration area and household sampling weights; ^B^18 households had 11–16 members; ^C^43 Living with a partner; ^D^Divorced, never married and widowed; and ^E^14 mothers had 10–14 parity history.

### Infants’ breastfeeding and complementary feeding practices

**Fig. 1:**
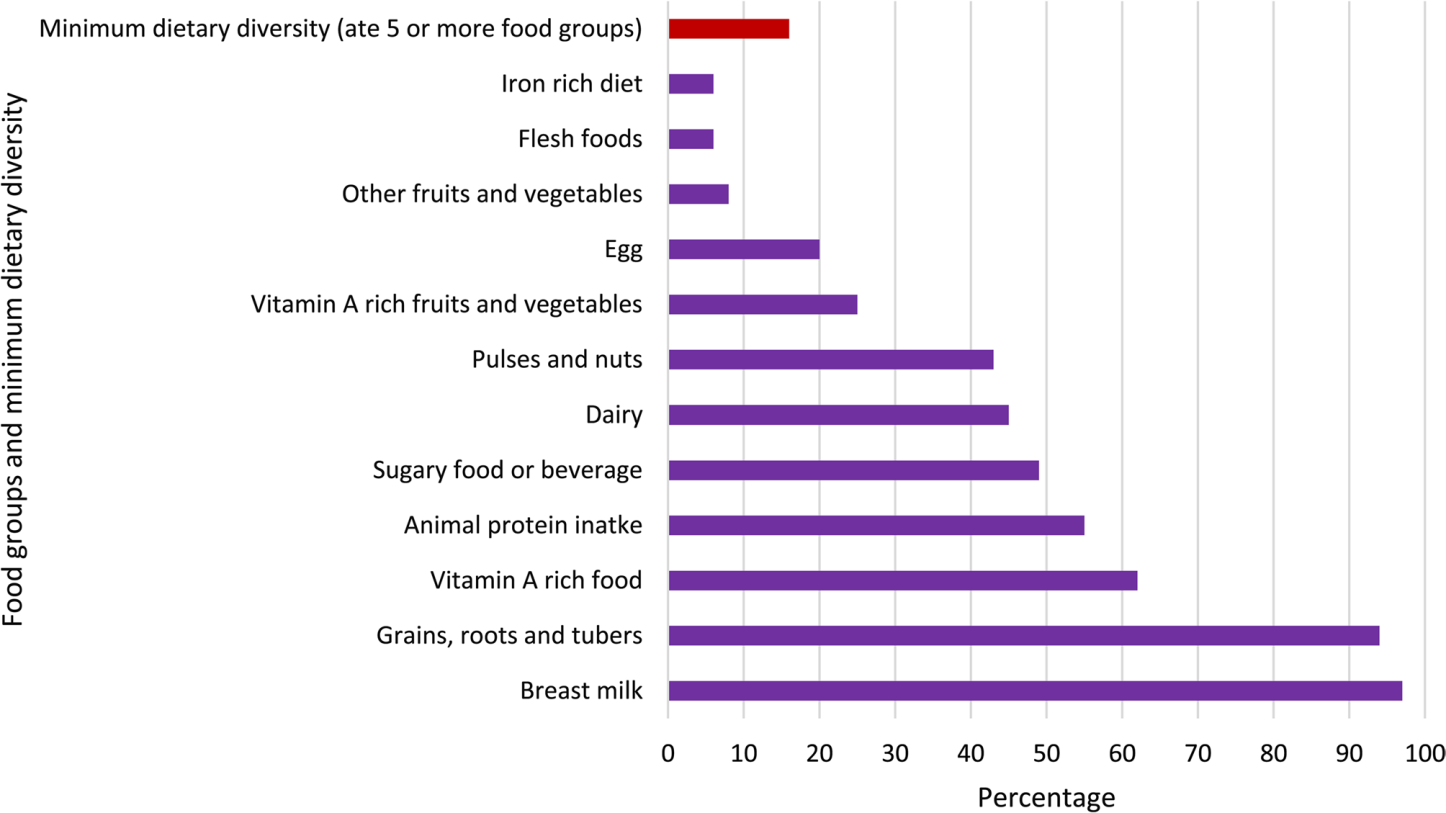
Fig. 1 Infants’ dietary diversity in Ethiopia, PMA panel study, July 2020 to August 2021 (n=1,850).

All infants started breastfeeding within one week, and 67% (95% CI 63, 71) were put to the breast within one hour of birth. A total of 69% (95% CI 67, 71) were exclusively breastfed at five months, and 97% were still being breastfed at 12 months. 16% of infants aged 12 months had consumed complementary food meeting the minimum diet diversity (Fig. 1). 62% had vitamin A-rich food during the past 24 h and 6% had consumed iron-rich foods. Half of infants (49%, 95% CI 44, 55) aged 12 months consumed sugary foods or beverages on the day before the survey.

### Socio-economic and educational equity in infants’ breastfeeding and complementary feeding

**Fig. 2:**
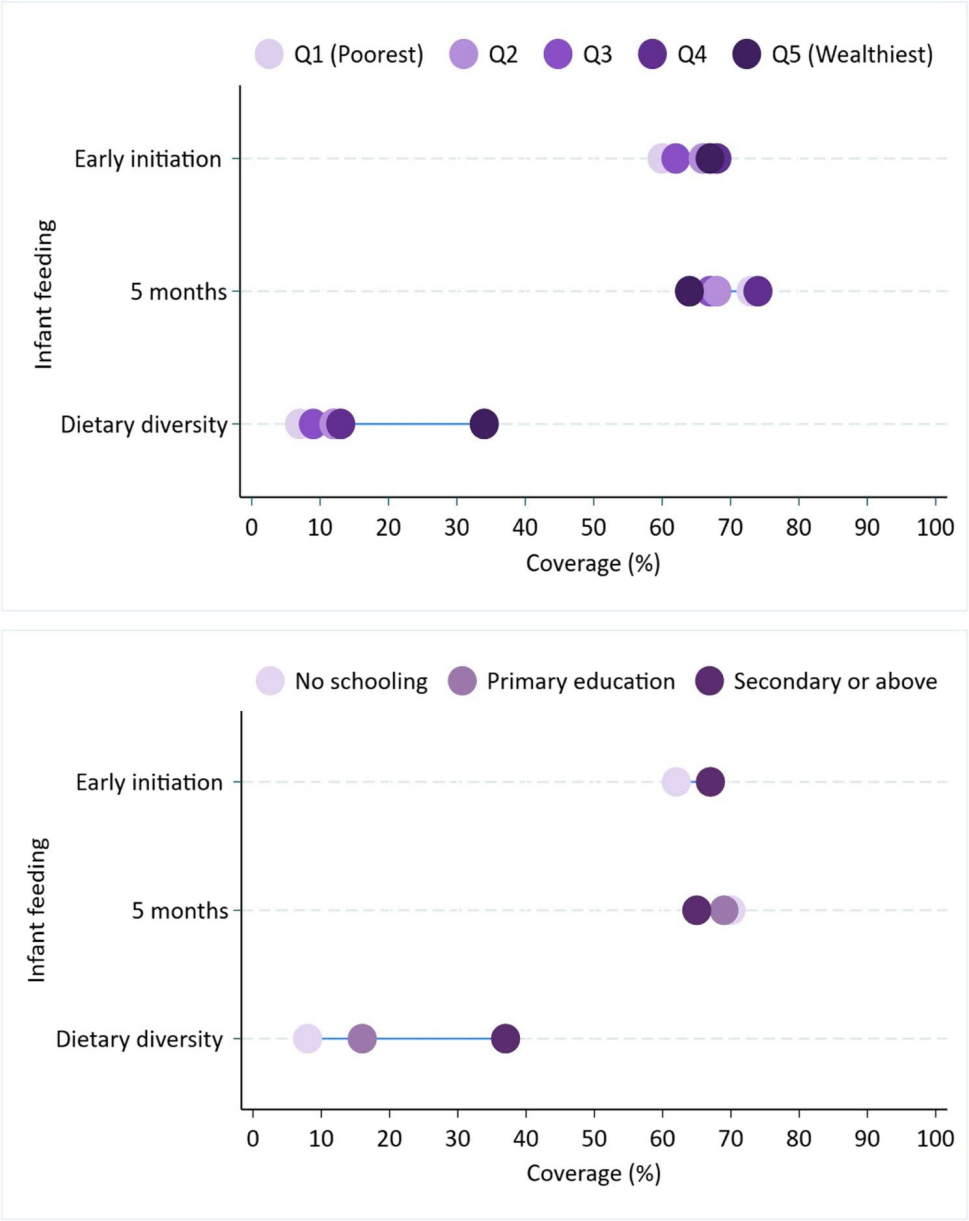
(**(a)**) Wealth- and (**(b)**) education-based social equity in infant feeding practices in Ethiopia, PMA panel study, July 2020 to August 2021 (n=1,850)

There was no evidence of inequity in infants’ early initiation of breastfeeding and exclusive breastfeeding at 5 months (Table 2; Fig. 2a and b). The absolute (p-value = 0.211) and relative (p-value = 0.410) equity analyses showed no evidence of wealth-based social inequity in infants’ early initiation of breastfeeding. Similarly, the slope (p-value = 0.171) and concentration index (p-value = 0.053) analyses showed an equitable distribution of exclusive breastfeeding by household wealth status. The slope of index analysis did not show any education-based inequity in infants’ early initiation of breastfeeding (p-value = 0.240) or exclusive breastfeeding at five months (p-value = 0.236) (Table 2). Early initiation of breastfeeding and exclusive breastfeeding at five months were not associated with household wealth and mothers’ education levels (Table 3). Mothers aged over 30 were less likely to initiate breastfeeding during the first hour than the youngest mothers (15–19 years). In contrast, early initiation of breastfeeding increased with parity.

**Table 2:** Wealth- and education-based inequities in infants’ inappropriate feeding practices in ethiopia, PMA panel study, July 2020 to August 2021 (*n* = 1,850)

Appropriate feeding practices	Overall appropriate feeding (%)	Q1^A^ appropriate feeding (%)	Q5^B^ appropriate feeding (%)	Difference (Q5-Q1; %)	Slope index of inequity			Ratio (Q5:Q1)	Concentration index		
					Coefficient (%)	SE	P-value		Coefficient (%)	SE	P-value
Wealth-based analysis											
Early initiation of breastfeeding	67	63	67	4	0.086	0.070	0.211	1.1	0.015	0.02	0.410
Exclusive breastfeeding at five months of age	69	71	63	−8	−0.079	0.058	0.171	0.9	−0.02.7	0.01	0.053
Minimum dietary diversity at 12 months of age	16	10	32	22	0.358	0.045	< 0.001	3.2	0.350	0.04	< 0.001
Education-based analysis											
	Overall appropriate feeding (%)	E1^C^ appropriate feeding (E1%)	E3^D^ appropriate feeding (E3%)	Difference (E3-E1; %)	Slope index of inequity			Ratio (E3:E1)			
					Coefficient (%)	SE	P-value				
Early initiation of breastfeeding ^E^	67	62	67	5	0.0738	0.062	0.240	1.1			
Exclusive breastfeeding at five months of age ^E^	69	70	65	−5	−0.0638	0.053	0.236	0.9			
Dietary diversity at 12 months of age ^E^	16	9	37	28	0.368	0.042	< 0.001	4.1			

^A^ Poorest

^B^ Wealthiest

^C^ No schooling

^D^ Secondary or above education

^E^ concentration index analysis was not employed as mothers’ education was a categorical variable (not a continuous variable)

**Table 3:** Social factors associated with early initiation and exclusive breastfeeding and minimum dietary diversity among infants who completed a one-year follow-up, PMA Ethiopia panel study, July 2020 to August 2021

Characteristics	Early initiation of breastfeeding^*^ (*n* = 1,843)			Exclusive breastfeeding at five months^*^ (*n* = 1,842)		Diet diversity at 12 months^*^ (*n* = 1,843)	
	Yes	COR^**^ (95% CI^#^)	AOR^***^(95% CI)	COR (95% CI)	AOR (95% CI)	COR (95% CI)	AOR (95% CI)
Household wealth							
Q1 (Poorest)	212	1.0	1.0	1.0	1.0	1.0	1.0
Q2	184	1.3 (0.7 to 2.3)	1.3 (0.7 to 2.4)	1.2 (0.8 to 2.1)	1.2 (0.7 to 2.1)	0.7 (0.3 to 1.4)	0.6 (0.3 to 1.3)
Q3	206	1.4 (0.8, 2.3)	1.4 (0.8 to 2.4)	1.1 (0.6 to 1.9)	1.2 (0.6 to 1.9)	1.2 (0.6 to 2.5)	1.1 (0.6 to 2.2)
Q4	224	1.1 (0.6 to 1.8)	1.0 (0.6 to 1.8)	1.1 (0.7 to 2.2)	1.1 (0.6 to 2.1)	1.8 (0.9 to 3.5)	1.5 (0.8 to 2.9)
Q5 (Wealthiest)	370	1.1 (0.6 to 1.8)	0.8 (0.4 to 1.5)	0.8 (0.5 to 1.4)	0.7 (0.3 to 1.7)	5.9 (3.3 to 10.7)	2.9 (1.4 to 6.4)
Education							
No schooling	467	1.0	1.0	1.0	1.0	1.0	1.0
Primary school	446	0.8 (0.6 to 1.1)	0.7 (0.5 to 1.0)	1.3 (0.9 to 1.8)	1.3 (0.8 to 1.8)	1.5 (0.8 to 2.4)	1.5 (0.9 to 2.7)
Secondary school or above	283	0.9 (0.6 to 1.4)	0.9 (0.6 to 1.5)	1.2 (1.7 to 2.1)	1.2 (0.7 to 2.1)	4.5 (2.7 to 7.3)	3.7 (1.9 to 7.0)
Mothers age							
15–19 years	97	1.0	1.0	1.0	1.0	1.0	1.0
20–24 years	299	1.1 (0.7 to 1.9)	0.9 (0.5 to 1.6)	1.0 (0.6 to 1.7)	0.9 (0.6 to 1.5)	0.9 (0.5 to 2.2)	0.8 (0.4 to 1.8)
25–29 years	294	0.9 (0.6 to 1.7)	0.6 (0.3 to 1.2)	0.9 (0.5 to 1.5)	0.8 (0.4 to 1.4)	1.3 (0.7 to 2.4)	0.9 (0.5 to 2.0)
30–34 years	225	0.8 (0.5 to 1.3)	0.4 (0.2 to 0.8)	0.9 (0.5 to 1.5)	0.9 (0.5 to 1.5)	1.3(0.6 to 2.8)	1.2 (0.5 to 3.1)
35–47 years	181	0.8 (0.5 to 1.5)	0.4 (0.2 to 0.9)	0.7 (0.4 to 1.3)	0.8 (0.4 to 1.6)	1.19 (0.5 to 2.9)	1.3 (0.4 to 4.1)
Parity^&&^							
1	197	1.0	1.0	1.0	1.0	1.0	1.0
2	280	1.1 (0.8 to 1.7)	1.4 (0.9 to 2.1)	1.4 (0.9 to 2.1)	1.6 (0.9 to 2.4)	1.7 (1.1 to 2.8)	1.9 (1.1 to 3.2)
3–4	371	1.4 (0.9 to 2.0)	2.1 (1.3 to 3.4)	1.4 (0.9 to 2.3)	1.7 (1.0 to 2.7)	1.1 (0.7 to 1.8)	1.3 (0.8 to 2.3)
≥ 5	345	1.2 (0.8 to 1.7)	2.4 (1.3 to 4.2)	0.9 (0.6 to 1.6)	1.2 (0.7 to 2.3)	1.1 (0.6 to 1.8)	1.7 (0.8 to 3.4)

* Analysis adjusted for mothers’ place of residence, place of delivery, and antenatal care

** Crude Odds Ratio

*** Adjusted Odds Ratio

# Confidence Interval

**Fig. 3:**
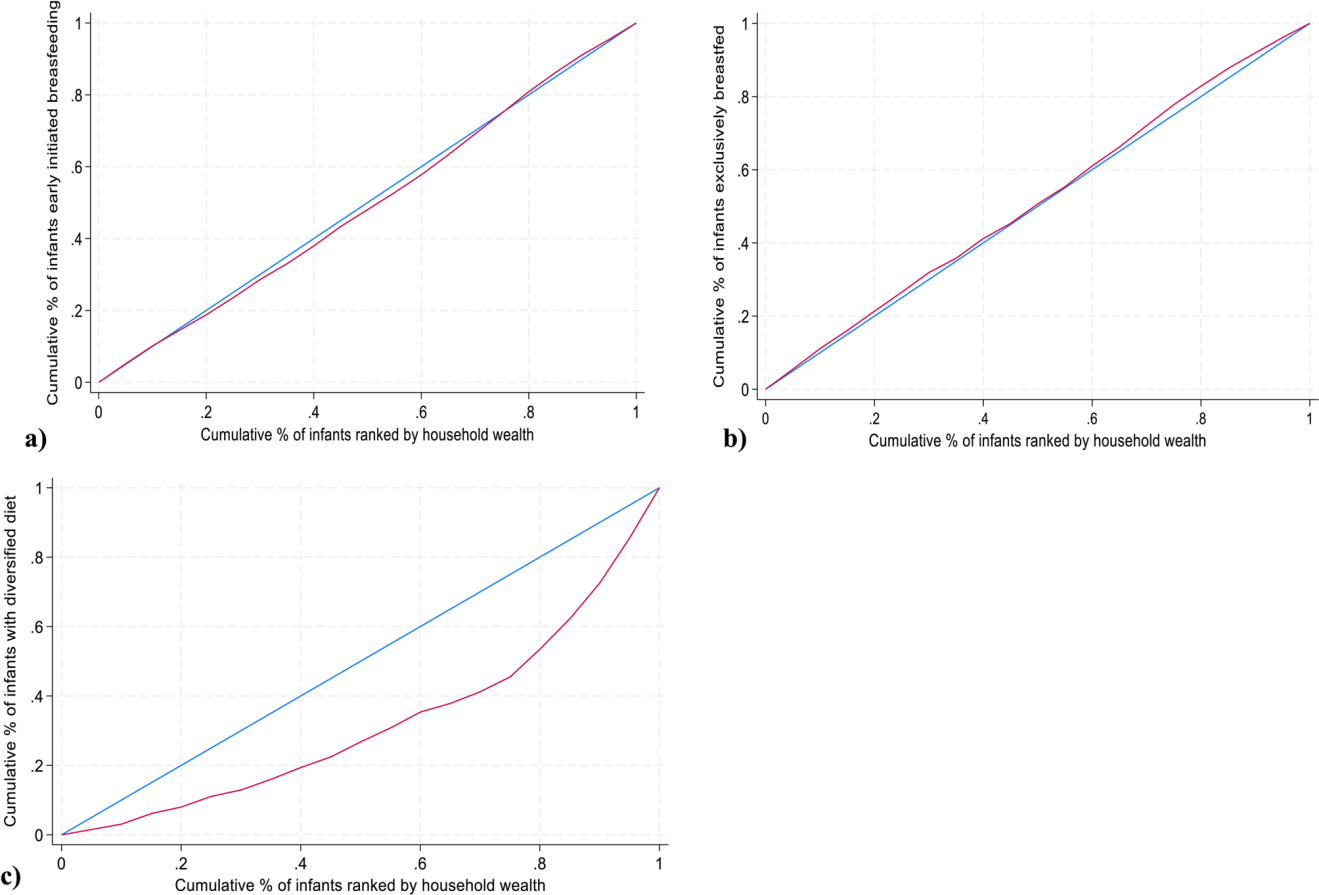
Concentration curve for (**a**) early initiation of breastfeeding, (**b**) exclusive breastfeeding at five months of age, and (**c**) dietary diversity at 12 months by household wealth in Ethiopia, PMA panel study, July 2020 to August

The absolute and relative equity analyses showed wealth-based variation in infants’ diet diversity (Table 2; Figs. 3 and 2c). In an absolute measure, compared with the lowest household wealth group, the highest wealth group had a higher proportion of infants with minimum diet diversity. The slope (0.358) and concentration (0.35) index coefficients showed considerable gaps in diet diversity, favoring the infants from better-off households. Similarly, in a relative measure (Q5: Q1; 3.2) the likelihood of minimum dietary diversity was higher in better-off households.

The proportion of infants fed complementary food at 12 months with at least minimum-level dietary diversity was also considerably higher among infants of educated mothers (37%) compared to those without schooling (9%), resulting in an absolute inequity (E3-E1) of 28%. The percentage of infants with minimum dietary diversity among infants from mothers with no schooling and better-off families was 16%. The slope index of 0.368 showed a wide gap in infants’ diet diversity between educated and less educated mothers. The likelihood of minimum diet diversity was 4.1 (E3:E1) times higher among infants of educated mothers compared to mothers without schooling.

Infants from the wealthiest households compared with the least wealthy [AOR = 2.9; 95% CI: 1.4 to 6.4] and infants with mothers having at least secondary school education compared with no education [AOR = 3.7; 95% CI: 1.9 to 7.0] were much more likely to receive complementary foods from at least five food groups when they were one year old, Table 3.

## Discussion

The high coverage of initiation of breastfeeding within one hour of birth and exclusive breastfeeding at five months of age was equitably distributed by mothers’ education and household wealth in Ethiopia. However, at 12 months of age, most infants had poor-quality complementary food. Infants’ quality of complementary food showed significant pro-rich and pro-educated inequity, implying that it favored socially advantaged families.

This study is the first to provide comprehensive insight into social equity in infants’ breastfeeding and complementary feeding practices in Ethiopia. The coverage of infants’ breastfeeding and the quality of complementary foods were assessed among infants sampled from five administrative regions representing most (85%) of the Ethiopian population [20]. We prospectively measured infants’ breastfeeding from birth to one year, reducing recall bias. Measuring infants’ dietary diversity using 24-hour recalls implies accurate reporting of the current food consumption on the group level. However, using a single 24-hour recall has the limitation of not showing the individual’s “usual” dietary habits [19].

Over two-thirds of infants had the recommended early breastfeeding initiation, and almost 70% were exclusively breastfed at five months, which is the current WHO target. This coverage was higher than reports from other low- and middle-income countries for early initiation and exclusive breastfeeding at five months of age [13]. The early initiation and exclusive breastfeeding coverage was consistent with the 2019 Ethiopia Demographic and Health Survey report [21]. Our findings showed equitable distribution of early initiation and exclusive breastfeeding by mothers’ education and household wealth. In contrast, other low- and middle-income countries reported inequitable pro-educated and pro–rich distribution of appropriate breastfeeding [14, 15]. Ethiopia’s good breastfeeding culture and national breastfeeding efforts could explain the observed high coverage and equitable distribution of appropriate breastfeeding. Almost all (96%) Ethiopian infants’ started breastfeeding and, on average, breastfed for 23.6 months [21].

We found that only a tiny proportion of infants aged 12 months had complementary foods meeting the minimum quality criterion. A similarly low coverage of the minimum dietary diversity was also reported by the 2019 Ethiopia Demographic and Health Survey [21]. The 2022 UNICEF report showed that about half of Ethiopian children had severe food poverty, i.e., they had complementary food from one or two groups [22]. The reported prevalence of minimum dietary diversity was lower than the average estimates (25%) for sub-Saharan African and low- and middle-income (27%) countries [12, 23]. About 70 million children in sub-Saharan Africa lived in severe food poverty; one-tenth (8.2 million) of the burden was shown in Ethiopia [22]. The poor household food security or socio-economic status might explain the low quality of complementary food [24]. In Ethiopia, the primary livelihood strategy is traditional rain-fed agriculture combined with livestock husbandry. However, poor agricultural productivity is a major challenge, contributing to low annual per capita income and a high burden of household food insecurity [25, 26]. Livestock are primarily raised for sale to meet critical family expenses—including food, clothing, and healthcare—a practice that subsequently reduces the household’s consumption of animal products [27]. Consequently, this often means households consume fewer animal products, which contributes to poor dietary diversity. The traditional cereal-based monotonous diet remains a tradition in children’s complementary food preparation [21]. This cultural food choice may largely underline the poor quality of complementary food. The reported poor quality of complementary food could suggest infants’ higher risk of undernutrition, including micronutrient deficiencies [23, 28]. We also found that many infants consumed sugary food or beverages, indicating a poor quality of complementary food. Sugary food or beverage consumption harms children’s oral health, general health, and growth [29].

Our equity analyses showed that infants of better-off families had higher dietary diversity than their poorer counterparts. Despite the pro-rich social inequity, about two-thirds of the better-off families had poor-quality complementary food. A study of low- and middle-income countries also showed pro-rich inequity in infants’ and young children’s (6–23 months) dietary diversity [12]. A similar socio-economic disparity in the quality of complementary food was reported from sub-Saharan African countries [30]. The observed pro-rich social inequity in dietary diversity could be related to the negative impact of poor socio-economic status on household food security [31]. The poorest families have limited economic resources to access nutritious and varied food for children and families [32]. Ethiopia’s escalating food price inflation further poses a significant challenge for poor families to access nutritious food, specifically of animal food products and fruits [33, 34]. Most of the global burden of extreme poverty is found in South Asia and sub-Saharan African regions, including Ethiopia [35]. Strengthening the implementation of poverty reduction strategies or attaining the SDG target of ending extreme poverty is critical to ensuring household availability and consumption of nutritious food for children. Strengthening homestead farming, including chicken, vegetables, etc., and awareness-raising interventions to enhance household consumption of these products could further help to improve infants’ consumption of diversified or nutritious complementary food [36, 37].

Our findings showed pro-educated social inequity in infants’ dietary diversity. A multi-country study showed similar better infant dietary diversity with increased mothers’ education [38]. The reported association could be explained by the role of schooling in enhancing mothers’ access to health care, child nutrition counseling, and education services [39]. Reports from low- and middle-income countries showed that nutrition education and counseling increased mothers’ nutrition literacy and child dietary diversity [40, 41]. Mothers’ education has been vital in risk reductions for child undernutrition and survival [41–43]. Education improves employment opportunities, earnings, and household food security [44, 45]. Children’s dietary diversity was higher among employed mothers’ children compared to those of unemployed mothers [46].

## Conclusion

Most Ethiopian infants in our study initiated breastfeeding within one hour of birth and were exclusively breastfed for five months, with coverage consistent with the 2030 global breastfeeding targets. Infants’ early initiation and exclusive breastfeeding were equitably distributed by mothers’ education and household wealth. Most infants had a poor quality of complementary foods at 12 months, with little diversity of food. Half of one-year-old infants consumed sugary foods or beverages. Infants of wealthier households and educated mothers had better quality complementary food. Targeting socially disadvantaged families in the implementation of nutrition policy and programs promoting appropriate infant feeding is essential to enhance the quality of complementary food, i.e., dietary diversity and reducing sugary food and beverage consumption. Strengthening national efforts to reach the SDG target of ending extreme poverty could improve infants’ consumption of nutritious food. Reducing sugary food or beverage consumption may shift household’s expenses to diversified and healthy diets. Future investigations into the quantification of infants’ nutrient intake, employing repeated weighted or estimated weighed food record methods, would offer a more comprehensive understanding. This approach, by mitigating the limitations associated with the single 24-hour recall method utilized in the current study, would facilitate the identification of children highly susceptible to nutrient deficiencies and, consequently, the development of targeted feeding support strategies.

## Data Availability

All data used in this publication are publicly available at https://www.pmadata.org/data/request-access-datasets.
